# Editorial: HPV and Host Interaction

**DOI:** 10.3389/fcimb.2021.638005

**Published:** 2021-03-11

**Authors:** Domenico Mattoscio, Tarik Gheit, Katerina Strati, Assunta Venuti

**Affiliations:** ^1^ Department of Medical, Oral, and Biotechnology Science, University “G. d’Annunzio” Chieti-Pescara, Chieti, Italy; ^2^ Center for Advanced Studies and Technology (CAST), University “G. d’Annunzio” Chieti-Pescara, Chieti, Italy; ^3^ Infections and Cancer Biology Group, International Agency for Research on Cancer, Lyon, France; ^4^ Department of Biological Sciences, University of Cyprus, Nicosia, Cyprus

**Keywords:** cervical cancer, head and neck (H&N) cancer, HPV – human papillomavirus, non melanoma skin cancer, beta HPV types, alpha HPVs, HPV infection

Human papillomaviruses (HPVs) are a group of DNA viruses that infect basal keratinocytes in stratified mucosal or cutaneous epithelium. While the overwhelming majority of infections are asymptomatic, a subset causes a spectrum of diseases as a consequence of the benign or malignant transformation of the host cell.

After reaching the basal layer of the epithelium *via* micro-wounds and entering inside the target basal keratinocyte, the virus has the ability to exploit the differentiation program of the infected cell and usurp host cellular pathways to sustain viral life cycle. While most of the infections are efficiently cleared, some of them persist in the host, finally leading to disease progression.

More than 200 HPV types have been described so far and categorized in five genera (α, β, γ, µ, ν) based on their nucleotide sequence. The α genus contains HPV types that infect mostly mucosal and genital epithelia, including HPV16 and other high risk (HR) oncogenic viruses that are involved in cancer etiology. HR-HPVs are the causative agents of cervical cancer and are also associated with a number of other anogenital malignancies, such as those of the vagina, vulva, anus and penis. Importantly, HPV is also responsible for about 25% of head and neck (HNC) cases worldwide with rates that are steadily increasing, particularly for oropharyngeal cancer. Moreover, recent studies suggested a pro-tumorigenic role for some α-HPV types in colorectal and breast cancers, although the association between HPV and these tumors remains controversial.

The β, γ, µ, ν and a small number of the α-HPV types show tropism for cutaneous epithelia, usually inducing asymptomatic infections. Limited information is known regarding the consequences (if any) of these asymptomatic infections to host tissues. However, recent evidence suggested a role for cutaneous HPVs, in particular of the β genus, as contributor of non-melanoma skin cancer in synergy with other factors such as DNA damage, immunosuppression or genetic disorders. Therefore, despite the efficacy of the vaccination program designed to target several mucosal HPV types for cervical cancer prevention, HPV-driven tumors are still common and cause a huge social and economic impact. Thus, research in this area is of paramount importance.

In the first article of this Research Topic, Guion and Sapp provided a comprehensive description of the peculiar strategy used by HPV proteins to usurp Promyelocytic leukemia (PML) nuclear bodies (NBs) during viral infection. PML NBs are dynamic nuclear structures containing a variety of proteins, such as PML and Sp100, that crucially regulate a number of cellular functions. For this reason, PML NBs are frequently targeted by viruses to their own advantage. In their review, Guion and Sapp summarized evidence showing that, contrary to other viruses that disrupt PML NBs, HPV forces the PML protein to assemble a protective structure around the viral genome aimed to prevent immune clearance of viral particles. In addition, the review highlights that HPVs are able to delay Sp100 recruitment on viral genome thus preventing the restriction of HPV gene transcription in the initial phases of infection.

Next, Flores-Miramonte et al. reported the presence of β, γ, and unclassified HPVs in a cohort of 48 Mexican cervical cancer patients as detected by individual Next-Generation Sequencing (NGS) approaches. In particular, the authors found a surprisingly high rate of coinfection with the cutaneous β and γ types in cervical samples, raising the possibility that non-α-HPV types may have a role in cervical cancer progression. However, even if the oncogenic properties of cutaneous HPV are still a matter of debate, and despite the relative low number of samples analyzed, these findings could have important implications for both diagnosis and treatment of cervical cancer, widely considered as determined by mucosal α-HPVs so far.

To clarify the role of β-HPVs in carcinogenesis, Paolini et al. investigated the role of viral β-HPV15 E6 and E7 proteins in a model of incontinentia pigmenti, a rare genodermatosis characterized by alteration in IKKγ. Expression of viral proteins increased NF-kB activity and decreased apoptosis in IKKγ silenced keratinocytes. These results suggest that infection with β-HPVs in susceptible genetic backgrounds may stimulate tumor development by manipulating a key signaling pathway and influencing cell survival.

HPV-positive and HPV-negative HNCs differ in in etiology, pathology, and prognosis. Studying these distinct tumor entities at the HNC site provides an opportunity for basic understanding of carcinogenic process and may inform clinical guidance in the management of these diseases. In this Research Topic, two papers investigated molecular differences between these two cancer types. Citro et al. reported an increased expression of the oncogenic transcription factor ΔNp63α in a panel of HPV-positive HNC cell lines respect to HPV-negative cells. Aggarwal et al. discussed current evidence of host transcription factors’ role in HPV-positive and HPV-negative tumors in the attempt to recognize major contributors of HNC prognosis. The identification of specific biomarkers for HNC subtypes may help in driving specific therapeutic protocols and for prognosis.

Given the variety of HPV-driven human tumors, a thorough understanding of how the diverse viruses engage interactions with host cells is still missing. Indeed, while cervical carcinogenesis is well understood, less is known about the role of α-HPVs in HNC and β-HPVs in skin tumors. Intriguingly, the dual tropism of β- and γ-HPVs also opens important challenges to depict a clearer picture of HPV biology and indicates that unexplored possibilities for the diagnosis and treatment of HPV-diseases should be pursued.

The collection of articles in this Research Topic sheds some light on these issues, but also suggests that more efforts are needed to further clarify how HPVs exploit host cells during infection and transformation.

## Author Contributions

All authors contributed to the article and approved the submitted version.

## Funding

DM is supported by Fondazione Umberto Veronesi (FUV).

## Conflict of Interest

The authors declare that the research was conducted in the absence of any commercial or financial relationships that could be construed as a potential conflict of interest.

